# Abdominal Emergencies

**Published:** 1917-11

**Authors:** Stephen Y. Howell

**Affiliations:** Buffalo, N. Y.


### BUFFALO MEDICAL JOURNAL

Yearly Volume 73 NOVEMBER, 1917 Number 4

### ORIGINAL ARTICLES

The right is reserved to decline papers not dealing with practical medical and surgical subjects, and such as might offend or fail to interest readers. Contributors are solely responsible for opinions, methods of expression and revision of proof.

### Abdominal Emergencies.

By STEPHEN Y. HOWELL, A. M., M. D., M. R. C. S. Eng.

Before we enter upon the formal consideration of our subject it will be interesting and, perhaps, profitable to briefly summarize, by way of prologue, the remarkable fetal changes which terminate in the pericardial, pleural, abdominal and scrotal sacs as we know them in practice. Very early in its intrauterine life the body of the fetus contains a single compartment, or space, lying between the primitive gut and the rapidly growing body-wall. This cavity, the coelom, is lined with a thin layer of mesoblastic cells, or mesothelium, from which is later developed the serous membranes. High up in the cervical region, in close apposition to the overlying heart, is the septum transversum which receives its phrenic nerve from the adjacent cervical plexus.

As the remarkable migration from the neck to the thoracic bottom occurs, the phrenic nerve progressively lengthens; the septum transversum, now permanently located, is converted into the adult diaphragm by the muscular contributions from the dorsal and ventral plates, and the peritoneal cavity is established.

In like manner the testes migrate from the sites of the Wolffian bodies to their permanent scrotal compartments, drawing with them their nerves from the upper lumbar region and their serous tunics from the peritoneum. Pushing into the common coelum from behind as they develope, the heart and lung-buds are covered with the double serous folds which line the pericardial and pleural cavities.

Diverticula from the primitive intestine, archenteron or mid-gut are carried into the growing cephalic and caudal extremities of the fetus, forming the fore-and hind-guts respectively.

From the fore-gut are formed the pharynx, oesophagus, stomach, and the duodenum as far as the orifice of the common bile-duct. Distal to this point, the alimentary canal is differentiated from the mid-gut, up to the splenic flexure; while from splenic flexure to anus the hind-gut is represented.

In the abdomen, then, are associated the intestinal tract from lower oesophagus to rectum, with its budding glandular offshoots—liver and pancreas, as well as the more purely mesoblastic spleen and genitourinary organs. While some of these visceral tenants, the kidneys for example, are clothed merely on their ventral surfaces by the dorsal, or visceral, layer of the mesothelial sac, the primitive gut—a fraction of an inch in length—and its median dorsal mesentery, ranging freely forward, are at first completely invested.

And when we familiarize ourselves with the uniform rotations and displacements which Nature employs in her efforts to meet the growing demands for space of a digestive tube which lengthens some twenty feet, the mysteries of the peritoneum, which gloomed our early student-days, are satisfactorily solved.

That this particular crowded compartment, then, should afford a most fertile field for pathologic happenings, that it should prove the chief income-producer of the surgeon, are facts which should call for no surprise.

Consider the accidents and working-strains to which these abdominal organs are being constantly subjected! From the savage to the most gifted human, promptings of appetite and thrilling messages conveyed by the gustatory and olfactory nerves to appreciative cortical centers, despite the warnings of precept and experience, govern both the swallowing rate and the choice of ingesta. But, unless sterilized by heat or otherwise, each bolus of food introduces its quota of bacterial stowaways—some, doubtless, upon evil bent.

As the slated roofs of our homes protect from fire, so do their epithelial and endothelial investments defend our outer and inner body-surfaces, however involved may be Nature's architecture.

And, just as the absence of a single slate may imperil the home when threatened by flame, so may even microscopic epithelial defects, resulting from mechanical, chemical or necrobiotic agencies, extend the right hand of welcome to microbic intruders.

The wonder is that Nature's marvelous provisions should be at all adequate to the strain of keeping the digestive tube effectively at work under conditions seemingly so adverse. And yet the scriptural three-score-years-and-ten of maltreatment, injury and neglect to which the stomach and intestines are ignorantly subjected, even by the most enlightened of us, are survived by a surprising per cent. of mortals, and that, too, without any breakdowns of surgical importance. In like manner the strains and stresses undergone by the urinary and reproductive organs have filled many volumes of professional literature, though consequent lesions of vital import afflict but a small minority.

When the pathologic break does come, however, and the organic laboratory of frail flesh, yielding to adverse influences, evokes symptoms and conditions which rank the case as an "abdominal emergency," we are confronted with questions of the most serious import. Indeed, no class of cases call more imperatively for professional ability and strength.

Upon a correct diagnosis, followed by timely and proper treatment, hangs the issue—life or death. All of us will recall emergencies of this nature,—the wear-and-tear involved in the beneficent struggle, the buoyant satisfaction or horrid disappointment and depression evoked by the outcome.

Today, however, the technic involved in the preparation and operative treatment of abdominal emergencies is fairly uniform, and the time and space at our disposal forbid elaborate descriptions of details which are presumably, familiar to us all.

In the matter of diagnosis, however, a further brief trespass upon your patience and good nature may, I trust, be tolerated.

To correctly diagnose diseased conditions within the body-cavities is a goal toward which progressive medicine has ever struggled, and ability in this line always marks the leader. Why? Because it proclaims a general efficiency. It demands: first, mastery of the normal structure and functions of the body; then, a familiarity with the multiform changes in these incident to disease, together with their manifestations; and, finally, the ability to employ the most effective methods in locating the trouble. The pride of the illy equipped "snap-diagnostician" must necessarily be often humbled.

Dr. William J. Mayo is fond of relating, apropos of this, that during his earlier professional career, when he was in general practice, he felt perfectly qualified to diagnose, positively, any internal disorder; but, when it came to skin-diseases—well, he passed.

A patient who employs one of our profession rightly expects a correct diagnosis of his case and proper treatment. Should death result, despite the fulfillment of these conditions, no blame can properly follow.

How far short of this ideal fall the rank and file of internists and surgeons, however, is well shown by an article which appeared recently in the New York Times. It was the report of a committee of investigation, declaring that 47 per cent. of the autopsies made in the hospitals and dispensaries of New York City revealed diagnostic errors. Indolence and indifference, rather than incapacity, doubtless account for these startling figures—a record which is, I fear, not peculiar to the Metropolis.

Under abdominal emergencies may be grouped, we assume, all those pathological happenings, foreseen or unforeseen, which suddenly threaten life and demand immediate relief, medical or surgical.

As a rule, close concentration upon the history and clinical signs will reveal the nature of the trouble. But, again, after exhausting every diagnostic means, including X-ray and laboratory tests, one is forced not infrequently to clear up the mystery by an exploratory operation.

In November last a young married woman, aged forty-five, living in the country, experienced a few days of malaise attended with loss of appetite. Suddenly she was seized in the night with a severe chill, ''ached from head to foot,'' as she expressed it, vomited frequently; and, on the following day, black diarrhoeic stools of offensive odor began. Typhoid fever was suspected, as the water in the farm-well had been low; she was brought to a local hospital by her physician and committed to my care. When I saw her, on Tuesday afternoon, the abdomen was markedly tympanitic, temperature 102, pulse rapid and hard and patient decidedly septic. She informed me that after her initial chill she had suffered from a severe headache and general abdominal pains, the latter usually dull in character, most marked in the splenic region and relieved by each movement of the bowels. Moderately deep palpation revealed no unusual tenderness in the hepatic region, and but slight pain in the right lower quadrant of the abdomen. In the splenic area, however, pressure caused some pain, and the patient said that from the beginning this region had given her most distress. Widal and diazo reactions were negative; blood-count showed 23000 leucocytes, 93 per cent. of which were polymorphonuclears.

Pus was evidently present, but where? Three consultants were in the dark; but one finally declared his belief that pus would be found post mortem in the appendiceal region. No tumor could be felt; but, to clear up this point, I resorted to lumbar section under local anesthesia. Through an incision above the right iliac crest I dissected down to the subserous areolar plane, pushed the left index finger down behind the appendix, and explored directly and bimanually, but with negative result. When in doubt concerning the condition of the appendix, this procedure, by the way, has proved of great value to me in cases whose condition forbade any avoidable surgery.

On the following afternoon, three days after her entrance into the hospital, the patient died. The autopsy showed a normal appendix, several patches of enteritis in the left colon, just below the splenic flexure, no apparent disturbance of the extrahepatic biliary organs, but a liver simply bathed in free pus. A primary infectious colitis had evidently lighted up the metastatic perihepatic suppuration, via the portal vein; and, under the circumstances, the lethal issue was excusable.

A certain number of abdominal cases, properly classed as emergencies, always have been, and probably always will be, unavoidable; but in the large majority of these it is delay, the so-called expectant treatment, that is directly responsible for fatal endings.

Acute obstruction of the bowels, for example, despite all the advance in technic which has marked the past thirty years, has a mortality but little changed for the better. Given a case exhibiting the classic diagnostic symptoms of pain, nausea and vomiting, the medical attendant is only too apt to run through the gamut of remedial measures. Buoyed up by the hope of final success and lured on only too often by the false symptomatic relief afforded by opiates and the soothing, insidious, fear-abating approach of that angel of death,—toxemia, the hours and days are allowed to pass. Suddenly the truth becomes but too apparent, the surgeon is summoned. Handicapped and protesting, he operates upon one practically dead, and another failure is checked up against his art.

In gastric ulcer, even with its grim potentialities, medical treatment is usually both justified and proper in the early stage, since healing frequently results. Duodenal ulcers, per contra, though healed will recur; and, after a period of suffering averaging twelve years, according to the late Dr. Murphy, surgery is resorted to.

As in appendicitis, operative interference is called for as soon as a duodenal ulcer is detected, and the first colic of a cholecystitis expresses a like demand.

In tubal pregnancy early operation puts a timely stop to the blind activity of the developing ovum and prevents the later hurry-up call of hemorrhage.

When finally nested in a follicle of the decidua serotina, the ovum sets about providing for its further placental maintenance through the phagocytic power of its external layer of syncytium. In like manner, when arrested en route in the Fallopian tube, the syncytial cells covering chorion and villi rapidly eat their way through the tubal tissues; and the eroded vessels, flooding the abdominal cavity, hoist the final peremptory signals of distress.

The early removal of pelvic tumors anticipates the dangers which threaten through malignant degenerations and twisted pedicles; indeed, few exceptions such as gastric ulcer can be adduced which favor delay in operative interference where experience has taught us that further evils are risked by such a course.

The five diagnostic methods of Dr. John B. Murphy* (*See Clinics, Vol. 1, No. 3, p. 459) are of great value to both internist and surgeon in the differential diagnosis of abdominal diseases. Those who are familiar with the great Chicago clinic will recall the frequent and enthusiastic references of that brilliant and talented teacher to these procedures, and his confidence in their findings. The results of trial and elimination during many busy years, they have proved their worth and can be confidently recommended to any practitioner whose self-confidence and success in this field are of the average order.

These methods are: (1) "fist percussion of the kidney," (2) "hammer-stroke percussion" in suspected disease of the gall-bladder or ducts, (3) "deep-grip palpation" of the gall-bladder, (4) "piano percussion" for the detection of small fluid exudates, and (5) "comparative bimanual examination" or simultaneous palpation of both the iliac fossae in suspected acute appendicitis.

Both gastric perforation and acute pancreatitis may be associated with pain, vomiting and collapse, varying in degree, but the absence of general abdominal rigidity and the early appearance of tympany favor the latter condition. It should further be borne in mind that jaundice evidences complete obstruction of the common duct, its duration and degree depending upon the length of the total occlusion; that a concretion causes colicky pain only when in motion, whether it be in the intestine, ureter or bile-passages; and, finally, that the abdominal cavity has frequently been opened for the relief of referred pain due to metastatic spondylitis or other spinal disorders.

When associated with pregnancy, appendicitis becomes a complication fraught with potential evil. It is a situation which may aptly be likened to a fire adjacent to a storehouse filled with explosives when pus forms about the appendix, and leads to a mortality which has been estimated at forty per cent.

Irritated directly and through its reflexes, the uterus is prone to contract and an abortion or miscarriage frequently follows. Hematogenous infection of the placental site is followed by a rapidly spreading metritis, septic phlebitis, embolic metastases and death.

When confronted with a case of appendicitis associated with pregnancy operate early, before pus has formed, or during a quiescent interval. Remove the inflamed appendix as quickly as posible, after a minimum exposure and handling of intestines, close the wound expeditiously and get your patient back in bed. Such a course will be least apt to interrupt gestation.

To determine the presence of pregnancy in doubtful cases calling for the operative relief of appendicitis or uterine fibroids, for example, the sero-diagnostic test of Abderhalden is of great value.

Though admittedly fallible, as are all tests, in fact, whether they bear the name of Widal, Wassermann or other laboratory expert; yet, a positive reaction is quite reliable, especially when corroborated by the history and clinical findings. The technic involved in its preparation and use, however, demands such delicacy and painstaking exactness that none save conscientious experts should be commissioned to make the test.

Abderhalden bases his reaction upon the assumption that a certain protein body is thrown into the blood of a pregnant woman by the syncytial chorionic cells, a product of their metabolism. Proteolytic to that particular cell-protein is an enzyme which is present in the blood-serum of the patient, produced somewhere in her body by the tissue-cells. This specific reaction of Abderhalden is a test for the presence of that enzyme in the blood-serum.

In cases of intestinal obstruction operative interference should be undertaken under local, rather than general, anesthesia whenever this is practical, and regardless of preparatory gastric lavage.

The longer the obstruction has persisted, the more imperative the rule. The stomach and those intestinal coils proximal to the point of obstruction in strangulated hernia, for example, are gradually filled with an astonishing amount of fluid—feculent, toxic and often offensive in character. When the patient is rendered incapable of intelligent action in his own behalf by general anesthesia, vomiting, should it occur, is very apt to result fatally. A prolonged flood of liquid material pours quietly from the mouth of the unconscious, weak and toxic victim; and assistance, however intelligent, is only too often unable to forestall the verdict: "drowned in his own vomit."

Finally, and apropos to this advice, Dr. Bevan, of Chicago, calls attention in a very recent article* (*Surgical Clinics of Chicago, Vol. 1, No. 1, p. 21) to an American synthetic, called "Apothesine," which is now being tested by 100 American surgeons before its general introduction to the profession of this country. It is said to excel novocain in merit. Used in the same strength, and with a like proportion of adrenalin as the latter German synthetic, it has proved to be non-toxic in amounts up to 150 c.c. and neutral in regard to healing. Since it can be boiled for five or ten minutes without deterioration, and produces anesthesia lasting about an hour, this new anesthetic bids fair to replace, in the near future, the beneficent contribution of that nation, the home of Kultur, which is now anathema, since she turned her face from the ways of righteousness to follow the devious paths of evil.

501 Delaware Avenue.

---

**Blood Pressure.** A. G. Browne, Jr., Charlotte Med. Jour., July, reviews the subject and gives the following standards: Under 1 year, 71 m.m.; under 1 year, 73 m.m.; under 2 years, 79.3 m.m.; under 3 years, 81 m.m.; under 4 years, 83 m.m.; under 5 years, 86.5 m.m.; under 6 years, 88.5 m.m.; under 7 years, 89 m.m.; under 8 years, 93 m.m.; under 9 years, 100 m.m.; under 10 years, 104 m.m.; under 12 years, 105 m.m.; under 20 years, (male), 120 m.m. (For any two years of age after this add 1 m.m. to 120 m.m.; so at 30 years, 125; at 60, 140.)

---

**Late Pregnancy.** R. G. Hann, Jour. of Obs. & Gyn., Sept. 1902, reported a woman who gave birth to her 13th child at the age of 49, three years after cessation of menstruation. M. E. Bove, Maple Ridge, Mich., Clin. Med., April 1917, reports a woman aged 53 in the 4th month of pregnancy. Her mother bore 25 children to 4 husbands, the last child at the age of 65. (Critic & Guide, June.)

---

**Caffeine Citrate in Strangulated Hernia.** Ortega, A Medicina Contemporanea, Feb. 4, employs this drug, 10 c.g. every quarter hour till 1 gram has been given, to stimulate contractility of the intestinal muscle.

